# The impact of maternal vitamin D levels during pregnancy and risk of autism spectrum disorder and attention deficit hyperactivity disorder diagnosis and symptoms in offspring: a systematic review and dose-response meta-analysis

**DOI:** 10.1007/s00787-026-03059-7

**Published:** 2026-06-03

**Authors:** Laura Bandini, Beatrice Vinceti, Teresa Urbano, Giuseppe Plazzi, Tommaso Filippini, Marco Vinceti

**Affiliations:** 1 Department of Biomedical, Metabolic and Neural Sciences, University of Modena and Reggio Emilia, Via Campi 287, Modena, 41125 Italy; 2 Department of Medical and Surgical Sciences (DIMEC), Alma Mater Studiorum, University of Bologna, Bologna, Italy; 3 Department of Biomedical and Neuromotor Sciences (DIBINEM), Alma Mater Studiorum, University of Bologna, Bologna, Italy; 4 Environmental, Genetic and Nutritional Epidemiology Research Center, Department of Biomedical, Metabolic and Neural Sciences, University of Modena and Reggio Emilia, Modena, Italy; 5 School of Public Health, University of California Berkeley, Berkeley, CA USA; 6 Department of Epidemiology, Boston University School of Public Health, Boston, MA USA

**Keywords:** Autism, Autism spectrum disorder, Attention deficit hyperactivity disorder, Vitamin D, Pregnancy, Neurodevelopmental disorders

## Abstract

Introduction: Autism Spectrum Disorder (ASD) and Attention Deficit Hyperactivity Disorder (ADHD) have a complex etiology involving both genetic and environmental factors, with mechanisms still unclear. In this review, we investigated the association between maternal 25-hydroxyvitamin D status during pregnancy and the risk of diagnosis and symptoms of ASD and ADHD in offspring in developmental age. Methods: We searched online databases up to February 13, 2026. We conducted a quantitative meta-analysis by comparing the exposure categories and using a dose-response approach across the entire exposure range. Results: We included 15 eligible studies: 8 related to ASD and ASD symptoms, 5 related to ADHD and ADHD symptoms, and 2 to both. Comparing high versus low maternal vitamin D status, we found an inverse association with both lower ASD risk (RR = 0.91; 95% CI 0.87–0.96) and ADHD risk (RR = 0.90; 95% CI 0.82–0.99). In the dose-response pooled analysis, ASD risk linearly decreased by 9% for every 10 nmol/L increase in maternal serum vitamin D levels. Higher vitamin D levels were also inversely associated with ADHD symptoms, though estimates were statistically imprecise due to the limited number of studies. Conclusions: Higher vitamin D status during pregnancy appears to be linearly associated with a reduced risk of ASD and ADHD diagnoses and symptoms in offspring, with no evidence of thresholds across the investigated exposure range. These findings suggest the opportunity to assess vitamin D levels during pregnancy, and to consider its supplementation in deficiency cases, to support fetal brain development and promote neuroprotection.

**Supplementary Information:**

The online version contains supplementary material available at 10.1007/s00787-026-03059-7.

## Highlights

Vitamin D acts modulating neuronal differentiation, synaptic plasticity, antioxidant effects, regulating inflammatory status, contributing to dopamine synthesis and signaling and immune homeostasis.

The findings indicate an inverse association between maternal/perinatal serum vitamin D levels and the risk of ASD, ADHD, ASD symptoms and ADHD symptoms in offspring of developmental age.

The linear dose-response analysis shows 9% reduction in the risk of ASD in offspring for every 10 nmol/L increase in maternal serum vitamin D levels.

Recognizing that these data represent only one of the factors and mechanisms that may contribute to the onset of ASD and ADHD, evaluating vitamin D levels during pregnancy and considering supplementation when appropriate could contribute to a neuroprotective effect on the brain development of offspring.

**Supplementary Information:**

The online version contains supplementary material available at 10.1007/s00787-026-03059-7.

## Introduction

The Autism Spectrum Disorder (ASD) and Attention Deficit Hyperactivity Disorder (ADHD) are common neurodevelopmental disorders characterized by high and rising global prevalence rates, with ASD reaching 1% in Europe, Asia and America and ADHD reaching prevalence of 7.6% among children aged 3 to 12 and 5.6% among teenagers aged 12 to 18 years [1, 2]. ASD diagnosis includes heterogeneous conditions that share impaired social interaction and restricted, repetitive patterns of behavior and interests. In particular, according to Diagnostic and Statistical Manual of Mental Disorders (DSM 5th-TR) these criteria must be met: (A) persistent deficits in social communication and social interaction across multiple contexts, present at time of diagnosis or in the past (as manifested by all the following behaviors: deficits in socio-emotional reciprocity, in nonverbal communicative behaviors used for social interaction, in the development, management and understanding of social relationships); (B) restricted and repetitive patterns of behaviors, interests or activities (as manifested by at least two of the following: stereotyped or repetitive movements, use of objects, or speech, excessive fidelity to routines, highly restricted and fixed interests that are abnormal in intensity or topic, hyper or hypo-reactivity to sensory input or unusual interests in sensory aspects of the environment); (C) symptoms must be present in the early development period, however they may appear later; (D) symptoms cause clinically significant impairment in social, occupational or other important areas of functioning; (E) the disturbances are not better explained by an intellectual developmental disorder or by a global developmental delay [3].

ADHD also encompasses a heterogeneous clinical presentation and is characterized by key symptoms such as inattention, hyperactivity and impulsivity: according to DSM at least six symptoms of inattention or six symptoms of hyperactivity/impulsivity must be present for at least six months before the age of 12 in two or more life contexts [3].

Both disorders have complex and multifactorial etiologies involving genetic and environmental factors. The genetic heterogeneity of the ASD phenotype is characterized by multiple loci and alleles involved in various biological processes [4, 5], an elevated genetic control [6], and by many prenatal factors that contribute to its onset [7–14]. ADHD, on the other hand, is characterized by a strong hereditary component [15] linked to genes that code for dopamine receptors and by an impaired dopaminergic signaling in the cortico-striatal circuits and by alterations in the maturation and function of dopaminergic neurons in circuits responsible for executive functions, response inhibition, and reward processing [16, 17].

In recent years, research has identified the contribution of mechanisms linked to immune, inflammatory and dopamine dysregulation in the genesis of these diseases [18–21].

Following the identification of the vitamin D receptors (VDRs) in both the fetal, neonatal and adult Central Nervous System (CNS) the role of vitamin D has become a focus of scientific interest [22, 23]. Throughout embryonic development, the VDRs are present in trophoblast, decidua, placenta, and various areas of the midbrain, as well as in the hippocampus, thalamus, hypothalamus, cortex, anterior cingulate cortex, amygdala and substantia nigra. The presence of VDRs in these key CNS regions, which are impaired in ASD and ADHD during embryonic development, suggests that vitamin D may act as a regulator of the developmental mechanisms involved in these disorders [24, 25]. With regard to ASD, vitamin D deficiency in the hippocampus, cerebral cortex, thalamus, and in the connections between the amygdala and the anterior cingulate cortex could result in abnormalities in the processing of sensory and social information [26]; whereas for ADHD, deficiency in the substantia nigra and midbrain could cause insufficient dopamine production, and deficiency in the cortex and striatum could cause difficulties in executive functions [27].

Vitamin D plays its key role in brain development by intervening in the regulation of many mechanism: neuronal proliferation and differentiation, connectivity, gene transcription, immune homeostasis, antioxidant mechanism and dopamine regulation [27–30].

Recent research highlights immune changes and low-level neuroinflammation in ASD and ADHD pathogenesis, affecting cerebral cortex development. In ASD, these changes lead to DNA modifications and gene expression shifts, causing molecular irregularities and altered immune responses. Observations include abnormal cell migration, synaptic pruning, and elevated pro-inflammatory markers such as TNF-alpha and IL-6 in the CNS, microglial and astroglial activation, elevated peripheral active B and NK cells, an elevated Th1 cell responses and reduced Th2 associated responses, disrupting immune homeostasis [19, 20]. Vitamin D is thus crucial for immune balance, especially in regulating Th1-driven autoimmunity. Deficiency leads to increased Th1 responses and decreased Th2 responses, linking vitamin D intervention efficacy to reduced Th1 activity and enhanced Th2 cell induction [30].

ADHD neuroinflammation involves pro-inflammatory cytokines and reduced cortical grey matter, impacting alterations in neurotransmitter systems including the dopaminergic, serotonergic and glutamatergic systems linked to executive functions and reward processing [16–18].

In particular, vitamin D promotes stem cell transformation into dopaminergic neurons and supports tyrosine hydroxylase expression, crucial for dopamine synthesis and neuron survival [27]. Deficiency in tyrosine hydroxylase limits dopamine availability, affecting executive functions in ADHD, while vitamin D regulation of dopaminergic, glutamatergic and GABAergic systems relates to synaptic imbalances in ASD [26]. Vitamin D also activates the transcription of the gene that codes for the enzyme tryptophan hydroxylase 2 responsible for the conversion of tryptophan into serotonin in the CNS. A deficiency of this enzyme compromises correct synaptogenesis and axonal growth, influencing social behavior, executive functions and impulsivity, and reflecting the picture present in ASD and in ADHD [31, 32].

The mother is the main source of vitamin D for the embryo: it crosses the placenta [33], and in studies on rats, whose brain development closely resembles that of humans, the presence of VDR in the neural epithelium has been observed as early as day 12 of embryonic development, as soon as the neural tube closes and cellular differentiation within the spinal cord begins [23]. Rats born to vitamin D-deficient mothers have been shown to experience pathological brain alterations at birth, specifically altered cell division rates, reduced expression of molecules vital for neuronal survival such Nerve Growth Factor (NGF) and Glial Cell Line-Derived Neurotrophic Factor (GDNF), with macroscopic brain structural changes [24].

Maternal vitamin D levels have been shown to be inversely associated with ASD and ADHD development in the offspring [34, 35] and have protective effects on language and behavioral deficits [36]. Some studies have suggested the occurrence of vitamin D deficiency in children affected by ASD and ADHD [37–39], and a beneficial effect of vitamin D supplementation on ASD symptoms and stereotypical behaviors [40], eye contact, attention span [41]. Such beneficial effects have also been suggested in ADHD, given the small improvements in total ADHD scores and in inattention, hyperactivity and behavioral scores following vitamin D supplementation in addition to methylphenidate [42].

Limitations of a recent review on the relation of vitamin D with ASD and ADHD [43] are the lack of uniformity in the diagnostic approach according to DSM or ICD criteria and standardized tests, and the outcomes represented categorical diagnoses, including non-homogeneous patient groups, such as those diagnosed with ASD and those with autistic symptoms, and those diagnosed with ADHD and those with ADHD symptoms. Therefore, in this systematic review and meta-analysis we aimed at assessing diagnoses based on DSM and ICD criteria and symptoms according to standardized tests. Furthermore, to reduce clinical heterogeneity and increase result validity, we divided participants from the included studies into four groups: subjects diagnosed with ASD, with ADHD, with ASD symptoms, and with ADHD symptoms.

We eventually aimed at defining the dose-response pattern of association between vitamin D levels and study endpoints across the entire range of exposure tested in the included studies. The hypothesis was an inverse association between disease risk and symptoms and serum vitamin D concentrations, based on the potential beneficial effects of vitamin D, as a molecule capable of promoting neuronal proliferation and differentiation, synaptogenesis, reducing pro-inflammatory states, and neurotransmitter regulation.

## Methods

In this review, we followed the Preferred Reporting Items for Systematic Reviews and Meta-Analysis 2020 guidance [44], adhering to PROSPERO database registration (International Prospective Register of Ongoing Systematic Reviews); Registry No: CRD4224569748.

### Search strategy and eligibility criteria

We performed a literature systematic search using MEDLINE (PubMed), EMBASE, Web of Science and The Cochrane Library databases, from inception up to February 13, 2026, without time restrictions. We used the keywords “vitamin D”, “autism spectrum disorder”, “attention deficit disorders with hyperactivity”, “ADHD”, “neurodevelopmental disorders”. The search terms included MeSH and non-MeSH terms, as reported in Supplementary Table S1.

We added the retrieved articles to the Rayyan QCRI online application for screening and selection process. We considered studies eligible when: (a) based on humans; (b) evaluated during developmental age (0 up to 17 years old); (c) with reported 25(OH)vitamin D blood concentrations during pregnancy (or, if not available, in newborns at birth); (d) assessing ASD, ASD symptoms, ADHD and ADHD symptoms evaluated by the Diagnostic and Statistical Manual of Mental Disorders (DSM) or by the International Classification of Diseases (ICD) and/or by standardized test scores; (e) written in English.

To ensure a comprehensive mapping of the vitamin D neurodevelopmental link, the inclusion criteria were extended beyond formal clinical diagnoses. We included studies evaluating: (1) the association between prenatal/neonatal vitamin D levels and the risk of diagnosis of ASD and ADHD; and (2) the relationship between vitamin D status and the presence of symptoms or neurodevelopmental traits in the pediatric population.

### Study selection and inclusion

**Fig. 1:**
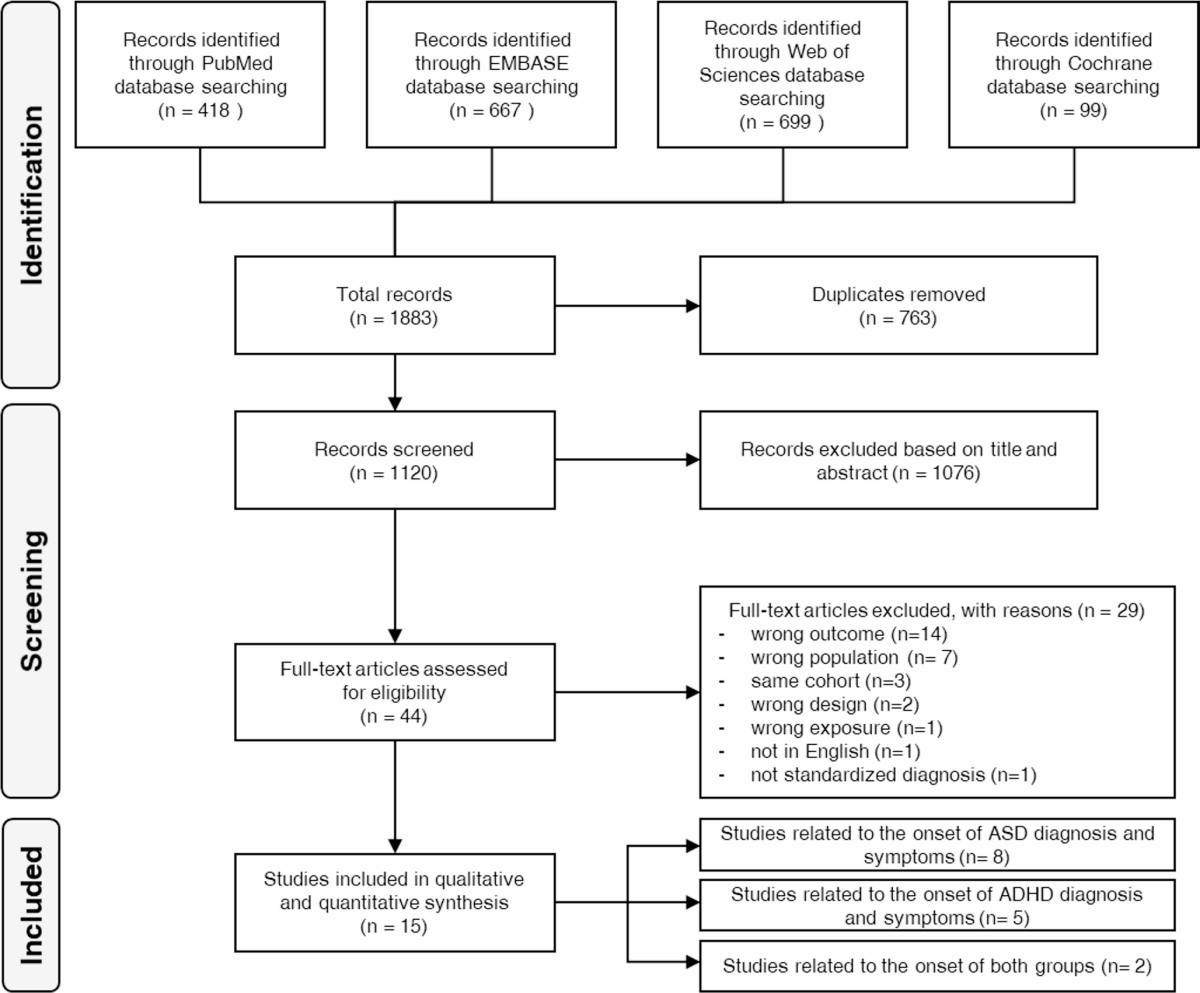
PRISMA flow-chart of the literature search

The selection process following PRISMA guidelines is presented in Fig. 1: we identified 1883 papers and after the removal of 763 duplicates, we excluded a further 1076 studies after title and abstract screening. After reading 44 full texts, we further excluded 28 studies mainly due to inadequacy of the outcome evaluated. We eventually included in the final analysis 15 papers: 8 studies related to the risk of diagnosis of ASD and ASD symptoms (5 case-control, 2 cohort and 1 case-cohort), 5 studies related to the risk of diagnosis of ADHD and ADHD symptoms (3 cohort and 2 case-control studies), and 2 studies for both groups (cohort studies).

### Data extraction

Three authors (LB, BV and TF) extracted the following data from each eligible study: first author, year of publication, country, study design, name of the cohort, years of mother recruitment (or newborn recruitment) and years of follow up and diagnostic assessment, aim of the study, population description, its sample number and number of patients with ASD and ASD symptoms, endpoint assessment methodology, vitamin D measurement method, timing of 25(OH)D levels assessment and 25(OH)D levels used. Vitamin D concentrations were expressed in nmol/L or, where necessary, converted from ng/mL into nmol/L. When several time points were available, we defined this preference list: preferring early pregnancy> late pregnancy > cord blood > blood spot at birth. Vitamin D level measurements at the beginning of pregnancy were preferred to provide greater homogeneity in exposure assessment among the included studies, since most of the measurements reported in the studies occurred in the first trimester and in the first weeks of the second trimester of pregnancy; secondly at 20 weeks; thirdly in the third trimester or at birth. We extracted the effect estimates including mean difference (MD) and the corresponding standard deviation (SD) or standard error (SE) for continuous outcomes, and odds ratio (OR), relative risk (RR), hazard ratio (HR), incidence rate ratio (IRR), and the corresponding 95% confidence intervals (CI) for dichotomous outcomes, and confounding factors. When results were presented using different statistical modeling, we extracted data for continuous linear vitamin D increase and from the most adjusted multivariable analysis. Any discrepancies among assessors were resolved through discussion with a fourth author (MV).

### Risk of bias assessment

We assessed risk of bias of the included studies using the Newcastle–Ottawa Scale (NOS) [45]. This scale includes three domains: the study-participant selection (0–4 scores), the comparability of study participants (0–2 scores), the exposure or outcome of studies (0–3 scores) for cohort/case-cohort and case-control studies, respectively, with a total score ranging from 0 to 9 scores, where 0–5 indicates a study of low-quality and 6–7 of moderate quality, and 8–9 of high-quality. Details of quality assessment are reported in Supplementary Table S2 for cohort/case-cohort and in Supplementary Table S3 for case-control design.

### Data analysis

We performed a meta-analysis using forest-plots comparing continuous linear exposure to vitamin D and disease risk or symptoms. We used inverse variance method to harmonized the categorical effect sizes (OR, HR, IRR) that provides a close mathematical approximation of the risk ratio because the primary outcomes evaluated are rare [46]. Similarly, for continuous effects (MD), since included studies implemented difference scales for symptom assessment, we used the standardized method estimating unitless MD [46]. Whenever possible, we evaluated the shape of the association between increasing vitamin D maternal levels and disease risk and symptoms using a dose–response meta-analysis based on the one-stage approach [47, 48]. To do this, we used the mean/median levels or the midpoint of each exposure category, depending on data availability, and if the highest and the lowest exposure boundaries were ‘open’, a 20% higher or lower value from the closest cut point [49, 50]. We carried out this analysis by using restricted cubic splines with three knots at fixed cut points (10th, 50th and 90th percentiles) and a restricted maximum likelihood random effects model using 25 nmol/L of vitamin D as reference [47, 48]. We also fitted a linear regression model reporting the slope of disease risk for continuous vitamin D increase alongside the non-linear relation.

We took into account the heterogeneity of results using I^2^ statistics in forest-plots and predicted study-specific curves in dose-response meta-analysis to assess variation across included studies [47, 51]. We also used funnel plots and Egger’s test to assess occurrence of publication bias [52] and trim-and-fill method to adjust the estimates in case of high risk [53] when at least five studies were available for the analysis. We used Stata software (v19.0, Stata Corp., College Station, TX, 2025) for all data analyses, namely with the ‘meta’, ‘mkspline’ and ‘drmeta’ routines.

## Results

### Characteristics of included studies

Table 1 reports the characteristics of the eight included studies concerning the association between maternal 25(OH)D levels and the risk of offspring ASD diagnosis during developmental age. These studies were published between 2016 and 2025 and were performed in China [34], Europe [54–58] and United States [59, 60].

**Table 1:** Characteristics of included studies in the systematic review related to maternal or perinatal vitamin D levels and the risk of ASD diagnosis in offspring

Reference	Country (Region)	Study design and cohort name	Recruitment and assessment period	Population	Sample (M/F)	ASD diagnosis	Time of circulating 25(OH)D levels assessment	25(OH)D (nmol/L) levels	Outcome	Adjustments factors
Chen 2016 [34]	China (Asia)	Case-control	Mothers between January 2014 -December 2015; children assessed at 3–7 years old (mean age 3.85 years)	Women with children diagnosed with ASD and an equal number of age and sex matched controls of women with typically developing children	Cases 68 (54/14) Controls 68 (54/14)	DSM-V	Maternal serum samples collected in the 1st trimester (11–13 gestational weeks)	< 39.4 39.4–47.9 47.9–57.2 ≥ 57.2 continuous per unit increase	OR 3.99 (2.58–7.12) 2.68 (1.44–4.29) 1.36 (0.84–2.58) 1.00 (1.00–1.00) 0.86 (0.80–0.91)	children’ age, gender, BMI and mothers’ age, BMI, gestational age at birth, tobacco use, gestational diabetes, gestational age at blood draw and serum levels of Hs-CRP, HCY, FA and Vit B12
Egorova 2020 [54]	Sweden (Europe)	Case-control (ASD children from the database of Child and Adolescent Psychiatry Department of Umea University, children with typically development from Northern Sweden Maternity Cohort)	Mothers who have children with ASD and with typically development born 1996–2009 (mean age not available)	women with an offspring diagnosed with ASD and an equal number of women with typically developing offspring matched for child’s sex, place and month of birth, year of blood serum sampling, mother’s age	Cases 100 (76/24) Controls 100 (78/22)	DSM-IV ICD-10	Maternal serum samples collected at the 1st midwife visit (between weeks 7 and 18, around week 14)	continuous by 1 SD increase	OR 0.77 (0.57–1.04)	mother’s age at sampling, year of sampling, sex of child, serum cotinine and mTHF levels
Horsdal 2025 [55]	Denmark (Europe)	Cohort (iPSYCH2012 case-cohort study)	children born between 1981–2005; assessed until 31 Dec 2012 (mean age not available)	ADHD children in sample with individuals with six mental disorders	Cases 8337 (nr) Overall sample 65,952 (nr) Childhood onset ASD+ADHD+Anorexia 27,476 (17610/9866)	ICD-10	Neonatal blood spots	< 16.79 16.79-21,44 21.44 − 25.61 25.61 − 31.00 > 31.00 Continuous per 1 SD increase	HR 1.06 (0.96–1.16) 1.02 (0.93–1.12) 1.00 (1.00–1.00) 0.91 (0.81-1.00) 0.84 (0.76–0.93) 0.93 (0.90–0.96)	age, sex, year of birth, birth month, maternal age, and parental history of any mental disorder
Lee 2019 [56]	Sweden (Europe)	Case-cohort (Stockholm Youth Cohort)	1996–2000 and screened for ASD from 1 to 60 months and then assessed (assessment mean age not available)	Maternal samples	Cases 449 (346/103) Controls 574 (305/269)	ICD-9 ICD-10 DSM-IV	Maternal serum samples collected at median 10.9 weeks gestation	< 25 25–49 ≥ 50 continuous for 25 nmol/L increase	OR 1.67 (0.76–3.67) 1.19 (0.79–1.78) 1.00 (1.00–1.00) 0.84 (0.60–1.16)	year of birth, sample month, and maternal characteristics (psychiatric disorders, age, BMI, smoking, nutritional supplement use)
Madley-Dowd 2022 [57]	United Kingdom	cohort (Avon Longitudinal Study of Parents and Children: ALSPAC)	births between 1 April 1991- 31 December 1992 (assessment at 3, 5, 7, 9 years)	mother-child pairs	Overall sample 5013 (nr)	ICD-10	maternal circulating in pregnancy, adjusted to 20 weeks for trimester two	Q1 29.3 Q2 42.2 Q3 58.9 Q4 75.6 Q5 114.1	OR 0.87 (0.42–1.80) 0.66 (0.31–1.41) 1.00 (1.00–1.00) 0.64 (0.30–1.37) 0.47 (0.21–1.06)	offspring sex, financial difficulties, maternal education, maternal occupational class, parity, maternal age at birth, pregnancy BMI and smoking status during pregnancy
Schmidt 2019 [59]	California (US)	Case-control (CHARGE case-control study)	Children aged 24–60 months enrolled and assessed (mean age 43.6 months ASD, 42.3 months TD)	Children with ASD and controls matched for state birth, catchment area distribution of autism cases, age and sex	Cases 357 (310/47) Controls 234 (191/43) Overall sample 725 (595/130)	ADOS ADI-R	Neonatal blood spots	< 50 50–74 ≥ 75 continuous for 25 nmol/L increase	OR 0.98 (0.63–1.51) 0.98 (0.66–1.46) 1.00 (1.00–1.00) 0.97 (0.87–1.08)	maternal education and pre-pregnancy BMI
Sourander 2021 [58]	Finland (Europe)	Case-control (FiPS-A study from the Finnish Maternity Cohort: FMC)	births between 1987–2004, with ASD diagnosis available in the Care Register for Health Care before 2015 (mean age at first ASD diagnosis 6.58 years)	Children diagnosed with ASD and in equal number matched controls for age and state of residence at diagnosis	Cases 1558 Controls 1558 Overall sample 3116 (2508/608)	ICD-8 ICD-9 ICD-10	Maternal serum samples collected at the 1st and early 2nd trimesters	< 20 20–39 40–59 60–79 ≥ 80 continuous for unit increase	OR 1.40 (1.05–1.87) 1.33 (1.02–1.75) 1.22 (0.93–1.59) 0.97 (0.75–1.26) 1.00 (1.00–1.00) 0.72 (0.59–0.89)	maternal age, gestational week of blood draw, season of blood collection, gestational age, maternal smoking, immigration, psychopathology and substance abuse
Windham 2020 [60]	California (US)	Case-control (Early Markers for Autism study: EMA)	children born between 2000–2003 (assessment mean age not available)	Children with ASD and population controls	Cases 534 (436/98) Controls 421 (349/72)	DSM-IV-TR	Maternal serum samples collected during mid-pregnancy (15–20 gestational weeks)	< 50 50–74 ≥ 75 continuous for 25 nmol/L	OR 0.79 (0.49–1.30) 0.93 (0.68–1.28) 1.00 (1.00–1.00) 0.95 (0.86–1.05)	birth year, blood draw season, child sex, maternal age, maternal education, maternal race/ethnicity and parity

*ASD* Autism spectrum disorder, *US *United States, *DSM-V* Diagnostic and statistical manual of mental disorders, 5th edition, *DSM-IV* Diagnostic and statistical manual of mental disorders, 4th edition, *DSM-IV-TR* Diagnostic and statistical manual of mental disorders, 4th edition, text revision, *OR* odds ratio, *BMI* body mass index, *ICD* International classification of disease, *ADHD* Attention deficit and hyperactivity disorder, *HR* hazard ratio, *BMI* body mass index, *ADOS* Autism diagnostic observation schedule, *ADI-R* Autism diagnostic interview-revised

Table 2 presents the features of the three included studies of the association between maternal 25(OH)D levels and ASD symptoms during developmental age. The studies were published between 2019 and 2025 and were conducted in Europe [25, 57, 61].

**Table 2:** Characteristics of included studies in the systematic review related to maternal or perinatal vitamin D levels and the risk of ASD symptoms in offspring

Reference	Country (Region)	Study design and cohort name	Period recruitment and assessment period	Population	Sample (M/F)	ASD symptoms evaluation	Time of circulating 25(OH)D levels assessment	25(OH)D (nmol/L) levels	Outcome	Adjustments factors
Lòpez-Vicente 2019 [61]	Spain (Europe)	population-based cohort (INMA Project)	between February 1997 and September 1998 in Menorca and between November 2003 and February 2008 in Valencia, Sabadell, Asturias and Gipuzkoa; (assessment at 5 years old)	mothers-child pairs	Cases 1510 Overall sample 2017 (1071/1036)	CAST at 5 years	maternal blood during pregnancy (mean 13.3 weeks of gestation)	< 49.9 49.9–74.6 ≥ 74.9 Continuous for 25 nmol/L increment	MD 0.00 -0.14 -0.26 -0.02	region, maternal age, maternal education level, maternal occupation, maternal country of birth, maternal smoking during the 1st trimester of pregnancy, partner smoking at home during the 1st trimester of pregnancy
Madley-Dowd 2022 [57]	United Kingdom	cohort (Avon Longitudinal Study of Parents and Children: ALSPAC)	births between 1 April 1991- 31 December 1992; (assessment at 3, 5, 7, 9 years)	mother-child pairs	Overall sample 7689 (4001/3688)	ICD-10 Autism factor mean score (AFM) calculated as the average of seven predictive factors for an autism diagnosis	maternal circulating in pregnancy, adjusted to 20 weeks for trimester two	Q1 29.3 Q2 42.2 Q3 58.9 Q4 75.6 Q5 114.1 Continuous for 10 nmol/L increase	MD 0.1 0.0 -0.0 -0.0 -0.0 -0.0	offspring sex, financial difficulties, maternal education, maternal occupational class, parity, maternal age at birth, pregnancy BMI and smoking status during pregnancy
van Rooij 2025 [25]	The Netherlands (Europe)	Population-based cohort (The Generation R cohort)	mothers enrolled between 2002–2006; (mean age at 13.46 years, range 9–11)	mother-child pairs children	Overall cases 3070 Overall sample 3937 (1951/1986)	SRS-2	serum levels in mid-pregnancy, median age of 20.36 weeks	Continuous for unit increase	Beta -0.07 (-0.12, -0.02)	age and sex

*ASD* Autism spectrum disorder, *regions: Asturias, Gipuzkoa, Menorca, Sabadell, Valencia,* UK* United Kingdom, *ADHD* Attention deficit and hyperactivity disorder, *CAST* Childhood autism spectrum test, *MD* mean, *ICD* International classification of disease, *SRS-2* Social responsiveness scale 2nd edition, *DSM-IV* Diagnostic and statistical manual of mental disorders, 4th edition, *AQ* Autism-spectrum quotient

In Table 3 are represented the characteristics of the four included studies of the association between maternal 25(OH)D levels and the risk of offspring ADHD during developmental age. The studies were published between 2015 and 2025 and were all performed in Europe [35, 55, 62, 63].

**Table 3:** Characteristics of included studies in the systematic review related to maternal or perinatal vitamin D levels and the risk of ADHD diagnosis in offspring

Reference	Country (Region)	Study design and cohort name	Period recruitment and assessment period	Population	Sample (M/F)	ADHD diagnosis	Time of circulating 25(OH)D levels assessment	25(OH)D (nmol/L) levels	Outcome (95% CI)	Adjustments factors
Gustafsson 2015 [35]	Sweden (Europe)	Case-control	births between 1978–2000 (assessment between 5–17 years)	children later diagnosed with ADHD and controls	Cases 202 (179/23) Controls 202 (161/41)	DSM III-R DSM-IV	cord blood	continuous for 0.1 ng/ml	OR 0.98 (0.95–1.01)	maternal age and smoking, season of birth
Horsdal 2025 [55]	Danish (Europe)	Cohort (iPSYCH2012 case-cohort study)	births between 1981–2005; children assessed until 31 Dec 2012 (mean age not available)	ADHD children in sample with individuals with six mental disorders	Cases 8844 (nr) Overall sample 65,952 (nr) Childhood onset ASD+ADHD+Anorexia 27,476 (17610/9866)	ICD-10	neonatal blood spot	< 16.79 16.79 − 21.44 21.44 − 25.61 25.61 − 31.00 > 31.00 continuous for 1 SD increase	HR 1.19 (1.08–1.311) 1.07 (0.97–1.18) 1.00 (1.00–1.00) 0.87 (0.79–0.97) 0.85 (0.77–0.94) 0.89 (0.86–0.92)	age, sex, year of birth, birth month, maternal age, and parental history of any mental disorder
Morales 2015 [62]	Spain (Europe)	prospective cohort (INMA Project)	first pregnancy visit between 1997–1998 in Menorca, November 2003-February 2008 in Valencia, Sabadell, Asturias, Gipuzkoa; (mean age 4.8 years)	mother-child pairs	1650 (820/830)	ADHD-DSM-IV	plasma concentration at 1st, 2nd and 3rd trimester; mean at 13 weeks of gestation	continuous for 25 nmol/L increment	RRR 0.87 (0.72–1.06)	area of study, child sex, child’s age at evaluation and maternal education level
Sucksdorff 2021 [63]	Finland (Europe)	case-control (CRHC)	births between 1998–1999; children assessed until 2011 (mean age 7.3 years)	1067 ADHD cases and 1067 matched controls	Cases 1067 (912/155) Controls 1067 (912/155)	ICD-10 code for Hyperkinetic disorder	maternal serum during the 1st and early 2nd trimester	7.5–21.9 22.0–27.6 27.7–36.4 36.5–49.4 49.5-132.5 continuous for one unit increase	OR 1.53 (1.11–2.12) 1.30 (0.94–1.79) 1.21 (0.89–1.65) 0.99 (0.73–1.33) 1.00 (1.00–1.00) 0.74 (0.58–0.93)	maternal age and maternal socioeconomic status and only for continuous maternal cotinine

*ADHD* Attention deficit and hyperactivity disorder, *DSM-III-R* Diagnostic and statistical manual of mental disorders, 3rd edition, revised, *DSM-IV* Diagnostic and statistical manual of mental disorders, 4th edition, *OR* odds ratio, *ICD* International classification of disease, *HR* hazard ratio, *RRR* Relative risk reduction

Table 4 displays the features of the four included studies of the association between maternal 25(OH)D levels and the risk of offspring ADHD symptoms during developmental age. The studies were published between 2015 and 2025 and were all conducted in Europe [25, 62, 64, 65].

**Table 4:** Characteristics of included studies in the systematic review related to maternal or perinatal vitamin D levels and the risk of ADHD symptoms in offspring

Reference	Country (Region)	Study Design	Period recruitment and assessment period	Population	Sample (M/F)	ADHD symptoms assessment	Time of circulating 25(OH)D levels assessment and 25(OH)D unit (nmol/L)	25(OH)D (nmol/L) levels	Outcome (95% CI)	Adjustments factors
Daraki 2017 [64]	Greece (Europe)	prospective cohort (the Rhea cohort)	between February 2007 – February 2008, 4 years follow up from October 2011 to January 2013 (assessment at 4 years)	mother-child pairs	487 (257/230)	ADHD-DSM-IV Hyperactivity inattention subscale of SDQ ADHDT	maternal serum 25(OH)D concentrations at the 1st prenatal visit (13 ± 2.4 weeks)	< 38.4 38.4–50.7 > 50.7	ADHDT 1.0 (1.00, 1.00) 0.96 (0.61, 1.54) 0.54 (0.33, 0.88)	child age of assessment, maternal age, parity, education, BMI pre-pregnancy, smoking during pregnancy
Morales 2015 [62]	Spain (Europe)	prospective cohort (INMA Project)	first pregnancy visit between 1997–1998 in Menorca, November 2003-February 2008 in Valencia, Sabadell, Asturias, Gipuzkoa (assessment mean age 4.8 years)	mother-child pairs	1650 (820/830)	ADHD-DSM-IV, ICD-10 ADHD symptoms inattention symptoms hyperactivity-impulsivity symptoms	plasma concentration at 13 weeks of gestation	ADHD symptoms < 49.9 49.9–74.9 ≥ 74.9 continuous for 25 nmol/L increase	IRR 1.0 (1.00, 1.00) 0.86 (0.63, 1.17) 0.77 (0.57, 1.04) 0.89 (0.80, 0.98)	area of study, child’s sex, child’s age at evaluation and maternal education level
Thinggaard 2024 [65]	Denmark (Europe)	Prospective cohort (Odense child cohort)	pregnancies between 2010–2012 (assessment at 5 years)	mother-child pairs	944 (509/435)	ADHD subscale score of CBCL for age 1.5-5 years	maternal serum in early pregnancy (before gestational week 20),	≤ 24 25–49 50–74 ≥ 75 continuous for 1 nmol/L increase ≤ 24 25–49 50–74 ≥ 75 continuous for 1 nmol/L change	OR 4.91 (1.27, 18.97) 1.32 (0.68, 2.57) 1.00 (1.00, 1.00) 1.09 (0.61, 1.94) 0.99 (0.98, 1.101) Beta 2.11 (0.85, 3.37) 0.22 (-0.25, 0.69 0.0 (0.0, 0.0) -0.23 (-0.63, 0.17) -0.01 (-0.02, 0.0)	maternal age, pre-gestational BMI, maternal educational level, parity, gestational age, birth season, parental psychiatric diagnosis, sex of child and children’s age at CBCL for age 1.5-5y
van Rooij 2025 [25]	The Netherlands (Europe)	Population-based cohort (The Generation R cohort)	Mothers between 2002–2006 (assessment at 9.67 years)	mother-child pairs	Overall cases 3334 Overall sample 3737 (1951/1786)	ADHD subscale score of CBCL for 6–18 years	serum levels at median gestational age of 20.36 weeks	continuous for unit/increase	beta -0.03 (-0.08, 0.02)	age and sex

*ADHD* Attention deficit and hyperactivity disorder, *DSM-IV* Diagnostic and statistical manual of mental disorders, 4th edition, *SDQ* Strengths and difficulties questionnaire, *ADHDT* Attention deficit hyperactivity disorder test, *ICD* International classification of disease, *IRR* Incidence rate ratio, *CBCL* Child behavior checklist, *OR* odds ratio

Participants in the studies included children of both sexes, aged from 0 to 17 years, for a total sample of 96,380 subjects. ASD and ADHD diagnoses were established using a variety of screening instruments, such as various editions of the ICD (ICD-8, ICD-9, ICD-10), and various editions of the DSM (DSM-III-R, DSM-IV, DSM-V, DSM-TR). Specific standardized tests were used for the diagnosis of ASD, such as the Autism Diagnostic Observation Schedule (ADOS), the Autism Diagnostic Interview-Revised (ADI-R), the Childhood Autism Spectrum Test (CAST), and the Social Responsiveness Scale 2 (SRS-2). Similarly, specific tests were used for the diagnosis of Attention Deficit and Hyperactivity Disorder, such as the ADHD-DSM-IV criteria, the Hyperactivity inattention subscale of Strengths and Difficulties Questionnaire (SDQ), the Attention Deficit Hyperactivity Disorder Test (ADHDT), the ADHD subscale score of Child Behavior Checklist (CBCL).

Vitamin D levels were assessed in the mothers’ serum during the first trimester of pregnancy for six studies, during the second trimester for seven studies and at birth via umbilical cord/spot blood for three studies.

### Results from the association between maternal vitamin D levels and ASD offspring

**Fig. 2:**
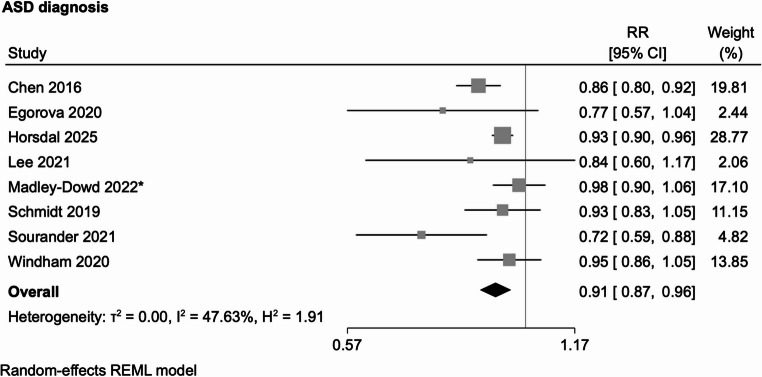
Forest plots of the studies related to the association between maternal vitamin D levels and offspring ASD diagnosis. RR: Risk Ratio, CI: Confidence Interval

The forest plot of eight studies (Fig. 2) indicates strong and consistent evidence of a protective association of vitamin D exposure to the risk of ASD. Despite the moderate heterogeneity (I^2^ = 47.63%), all studies reported lower risk with summary RR of 0.91 (95% CI 0.87–0.96). Funnel plot for publication bias (Supplementary Fig. 1) suggests possible asymmetrical distribution but the two added studies in the trim-and-fill analysis (Supplementary Fig. 2) did not substantially alter the summary RR (0.92, 95% CI 0.86–0.99).

**Fig. 3:**
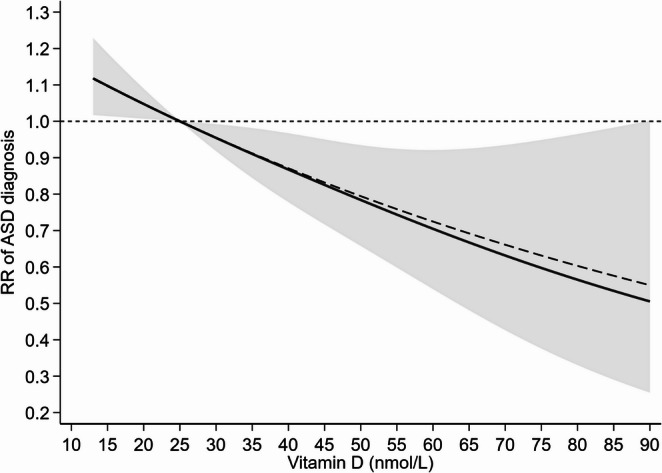
Dose-response meta-analysis according to increasing maternal vitamin D exposure concentrations and risk of offspring autism spectrum disorder (ASD). Spline curve is the combined estimate based on all studies (solid black line) with 95% confidence limits (grey area) and linear trend (dashed line). RR: Risk Ratio

The dose-response meta-analysis (Fig. 3) shows a substantial linear trend with decreasing ASD risk at increasing vitamin D levels, and linear regression trend indicates a RR of 0.91 (95% CI 0.83-1.00) each at 10 nmol/L of vitamin D increase. Study-specific dose-response curves (Supplementary Fig. 3) suggest a visual consistency across studies, with most trajectories showing a reduction in risk with increasing dose.

### Results from the association between maternal vitamin D levels and ASD symptoms offspring

**Fig. 4:**
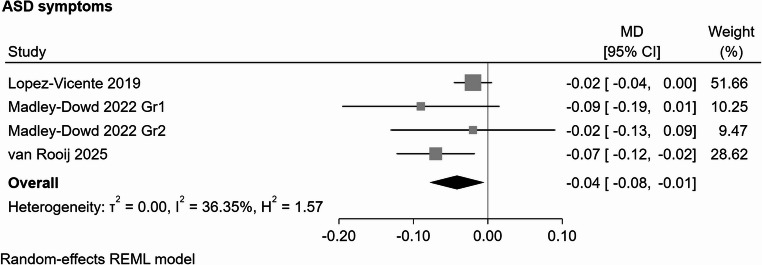
Forest plots of the studies related to the association between maternal vitamin D levels and offspring ASD symptoms. MD: Mean Difference, CI: Confidence Interval. For one study, Gr1 and Gr2 indicate two groups from the same publication with deficient and insufficient vitamin D levels, respectively

The forest plot of three studies concerning ASD symptoms (Fig. 4) indicates strong and consistent evidence of an inverse association between increasing vitamin D exposure and ASD symptoms in developmental age. Despite the limited number of studies hampering the assessment of publication bias and the low-moderate heterogeneity (I^2^ = 36.35%), all studies are in favor of decreased ASD symptoms with a pooled, overall MD of -0.04 (95% Cl: -0.08, -0.01).

### Results from the association between maternal vitamin D levels and ADHD offspring

**Fig. 5:**
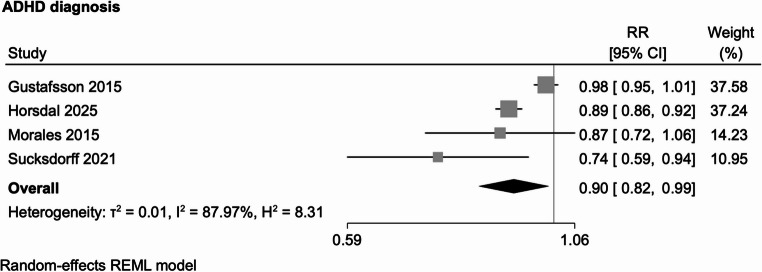
Forest plots of the studies related to the association between maternal vitamin D levels and offspring ADHD diagnosis. RR: Risk Ratio

The forest plot that correlates vitamin D levels with ADHD risk (Fig. 5) shows an inverse association between vitamin D levels, with a summary RR of 0.90 (95% CI 0.82–0.99), although with strong heterogeneity between the four available studies (I^2^ = 87.97%).

### Results from the association between maternal vitamin D levels and ADHD symptoms offspring

**Fig. 6:**
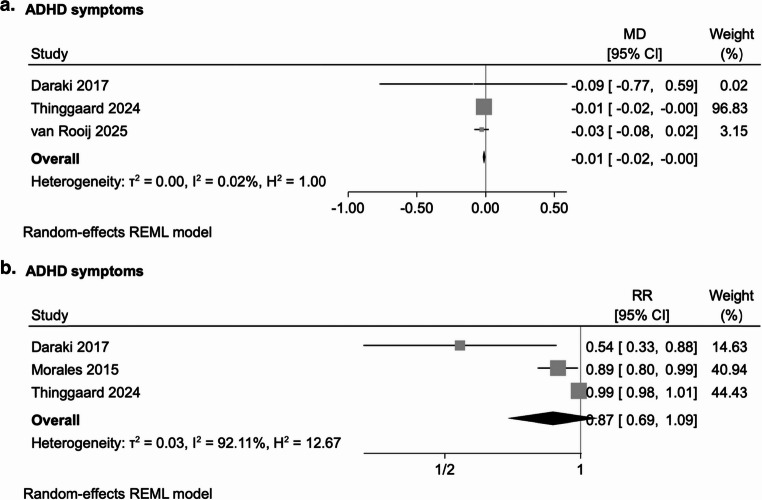
Forest plots of the studies related to the association between maternal vitamin D levels and offspring ADHD symptoms divided by modality of outcome evaluation, a: mean difference (MD), b: Risk Ratio (RR)

The forest plots for vitamin D levels with the onset of ADHD symptoms are presented Fig. 6, reporting respectively the association using continuous and dichotomous outcome. In the assessment considering continuous exposure, we grouped three studies, with a MD of -0.01 (95% CI: -0.02, -0.00), and excellent homogeneity between studies (I^2^ = 0.02%). In the dichotomous outcome assessment, we analyzed three studies, with a RR of 0.87 (95% CI: 0.69, 1.09) and high heterogeneity between studies (I^2^ = 92.11%).

In both analyses, indication of lower ADHD symptoms for increasing vitamin D exposure can be noted, although based on limited number of studies and therefore characterized by high imprecision of the estimates.

For both ADHD risk and symptoms, due to limited number of studies (< 5), we could not present funnel plot for publication bias.

## Discussion

In this review, we found an inverse association between maternal vitamin D serum levels and diagnosis and symptoms of both ASD and ADHD. The dose-response curve indicates a substantial linear trend toward a decrease in ASD diagnosis with increasing vitamin D levels and an approximate 9% reduction in the risk of ASD diagnosis in offspring for every 10 nmol/L increase in maternal serum vitamin D increase across the entire exposure range. The consistency of both outcomes towards a protective effect reinforces the robustness of our findings. Specifically, the diagnostic outcome reflects exceeding a conventional severity threshold and indicates that with higher vitamin D levels during pregnancy, fewer individuals accumulate sufficient symptoms to meet DSM/ICD diagnostic criteria for ASD and ADHD. The findings from the analysis of ASD and ADHD symptomatology as continuous endpoint suggest that higher vitamin D levels during pregnancy correspond to milder symptoms/behavioral phenotype in the population, regardless of whether a diagnostic threshold is exceeded or not. The potentially benefit on symptoms appears more sensitive, capturing even small changes, while the diagnostic effect appears clinically more robust but less sensitive.

The linear pattern of the dose-response relation between increased vitamin D intake during pregnancy and reduced ASD diagnoses in the offspring finds biological plausibility from the beneficial role of vitamin D for fetal brain development. The involved mechanisms could be regulation of neuronal proliferation and migration, immune and inflammatory modulation (by reducing the levels of pro-inflammatory cytokines in both the maternal gut and the fetal brain), and synthesis and balancing of dopaminergic, GABAergic, and serotonergic neurotransmitters and neurotrophic factors [27–30, 32]. 

Our dose-response curve indicated a lower risk of ASD diagnosis in offspring at concentrations above 25–30 nmol/L during pregnancy. This data confirms the scientific consensus and recommends maintaining circulating vitamin D levels above 25–30 nmol/L to avoid deficiency, broadening the scope to include neurodevelopment [66].

The observed differences in heterogeneity merit further discussion. The low-to-moderate heterogeneity of studies examining the link between maternal vitamin D levels and ASD diagnosis and symptoms in offspring could stem from differences among populations, study designs, and timing of circulating vitamin D measurement, as well as the different outcome assessment criteria over the years. The strong heterogeneity of studies examining the link between maternal vitamin D levels and the diagnosis of ADHD in children could derive from differences in the populations studied, study designs, diagnostic criteria used over the years, timing of circulating vitamin D measurement, and age of assessment. About the relation between vitamin D levels and ADHD symptoms, we found strong consistency across studies regarding the assessment of continuous outcomes, whereas a significant degree of variability emerged for the assessment of dichotomous outcomes. This could be mainly due to differences between populations at the geographical and sociocultural levels, recruitment periods, and differences in statistical methods. Due to the limited number of studies, we could not explore such heterogeneity in greater depth, hindering the implementation of further stratified analyses.

ASD and ADHD are complex neurodevelopmental disorders with a multifactorial etiology that includes genetic and environmental causes and mechanisms that are still unknown. Regarding the pathophysiology of these conditions, recent studies have identified the role of immune alterations and chronic low-grade chronic neuroinflammation as key pathogenic mechanisms in both ASD and ADHD that may interfere with cerebral cortex development [67]. It is thought that, in ASD, particularly before and after birth during critical periods for brain development, these factors may cause alterations such as DNA methylation, histone methylation and acetylation, altered splicing and changes in gene expression in the brain transcriptome, resulting in molecular abnormalities [68]. Studies demonstrate altered cell migration and synaptic pruning defects, loss of Purkinje cell layer and granular cell layer neurons, an increased innate and adaptive immune response through the Th1 pathway and increased peripheral active B and NK cells in ASD, which disrupts immune homeostasis [19]. There is an increased pro-inflammatory state and a cytokine storm such as TNF-alpha, IL-6, GM-CSF, IFN-gamma, IL-8 in the Central Nervous System (CNS), and marked activation of microglia and astroglia in the cerebellum and in the white matter of the autistic brain [20, 69]. Animal studies report this loss of immune homeostasis associated with behavioral abnormalities [70]. Neuroinflammation also plays an increasingly recognized role in the pathogenesis of ADHD and seems responsible for increased pro-inflammatory cytokines in cerebrospinal fluid and peripheral blood samples [71], reductions in the volume of cortical gray matter and certain cortical areas, and alterations in neurotransmitter systems including the dopaminergic, serotonergic and glutamatergic systems [16–18]. ADHD is, in fact, characterized by impaired dopaminergic signaling in the cortico-striatal circuits responsible for executive functions, response inhibition, and reward processing, structural changes such as cortical thinning and disrupted connectivity, and animal studies confirm neuroinflammatory and immune system alterations [72].

The identification of vitamin D receptor (VDR) expression in many brain regions, particularly in critical areas to both disorders (prefrontal cortex, limbic system, basal ganglia, forebrain, connections between the amygdala and the anterior cingulate cortex, and cerebellum) has focused attention on effects of vitamin D on maintaining inflammation control, as well as immune and neurotransmitter balance in CNS and on its potential involvement in ASD and ADHD pathophysiology [22].

Vitamin D acts as a crucial neurosteroid and exerts its action by modulating neuronal differentiation [27], synaptic plasticity [73], antioxidant effects [29], regulating inflammatory status [74], contributing to dopamine synthesis and signaling [27] and immune homeostasis [30, 75]. Vitamin D induces transcription factors that promote the transformation of stem cells into dopaminergic neurons, express tyrosine hydroxylase (the key enzyme responsible for dopamine synthesis in the substantia nigra), and allow the release of growth factors that ensure the survival of dopamine-using neurons. A deficiency of the enzyme tyrosine hydroxylase reduces the bioavailability of dopamine impacting on the fronto-striatal circuits responsible for executive functions, as manifested in ADHD [27]. Vitamin D also regulates the balance between the dopaminergic, glutamatergic and GABAergic systems and its deficiency is associated with a lower expression of GABA and glutamate transporters, contributing to the synaptic imbalance typical of autism [26]. Vitamin D also activates the gene transcription that encodes the enzyme tryptophan hydroxylase, which is essential for converting tryptophan into serotonin in CNS. A lack of this enzyme disrupts proper synaptogenesis and axonal growth, affecting social behavior, executive functions, and impulsivity, which are characteristics found in ASD and ADHD [31, 32].

The pivotal role of vitamin D in immune homeostasis is most evident in its capacity to control Th1-driven autoimmunity, whereby hosts deficient in vitamin D or VDR exhibit elevated Th1 cell responses and reduced Th2-associated responses [73], which corresponds to the condition observed in the brains of postmortem autistic patients [19]. The findings support a model where vitamin D based interventions derive their efficacy from the targeted inhibition of Th1 cell counts and function and from the compensatory induction of other CD4 + lineages, such as Th2 cells.

The main circulating form of vitamin D is 25(OH)D, and it is the best biomarker for monitoring vitamin D status. There is a general agreement that deficiency corresponds to levels below 25–30 nmol/L, inadequacy to serum concentrations from 30 to 50 nmol/L, and sufficiency to concentrations above 50 nmol/L [66, 76, 77], however cut-offs are now beginning to be considered less rigid in the healthy population. The evidence instead suggests significant clinical benefits of targeted empirical supplementation in specific populations (pregnant women, children, the elderly, individuals with prediabetes). In particular vitamin D deficiency during pregnancy is a global health issue, affecting over 54% of pregnant women, and it is related to fetal and neonatal levels [78]. According to recent guidelines, a vitamin D supplement is recommended for pregnant women to lower risk of preeclampsia, intrauterine mortality, preterm birth, small for gestational age birth, and neonatal mortality and for children aged 1 to 18 years in order to prevent nutritional rickets and reduce the risk of respiratory tract infections [79]. However, recent findings have also shown a role of vitamin D in neurodevelopment, demonstrating that vitamin D levels are significantly lower in children with ASD and ADHD [80, 81], that lower levels are related to more severe behaviors [82] and that supplementation reduces the core and behavioral symptoms in ASD [40] and in ADHD [83].

Our findings confirm the association between maternal/perinatal vitamin D levels and ASD and ADHD in offspring, expanding on the case series of a previous review [43]. Compared to work by colleagues and in relation to the newly published studies, three studies were excluded from the analysis because their cohorts were the same of other studies [61, 84, 85], while two studies were excluded because they did not meet the eligibility criteria regarding standardized outcomes and age [86, 87].

Among the limitations of this review, the large prevalence of data from European cohorts reduces the external validity of the results for other populations. The high prevalence of vitamin D deficiency globally is driven by many factors including high latitude, seasonality, female gender, socioeconomic status, geographic region, skin color, and cultural practices. Yet, a recent analysis of about 8 million participants confirmed vitamin D deficiency as a global health emergency, with nearly 15% of the worldwide population experiencing severely deficient levels (< 30 nmol/L) and around 48% experiencing insufficiency (< 50 nmol/L) [88]. The wide variability in findings is further accentuated by a marked geographical imbalance of studies, most of which were conducted in Western countries such as Europe and the United States [88], thus hampering a comprehensive assessment of the relation between vitamin D status and prevalence of neurodevelopmental disorders such as ASD and ADHD.

In addition, some summary estimates of our pooled analysis were statistically imprecise due to the small number of included studies, particularly concerning ADHD, thus hampering the implementation of further stratified analysis to explore the considerable heterogeneity of some results. Furthermore, considering that ADHD and ASD have a hereditary component and are associated with low vitamin D concentrations, the results could reflect such overall genetic association rather than support a protective role for vitamin D, also since most studies did not adjust for parental diagnoses. In fact, only four of them considered maternal diagnoses, and only one also considered paternal diagnoses.

A further limitation of this systematic review and meta-analysis is the variability across included studies in exposure assessment, particularly measurement methods and timing. In addition, and despite the adjustment in data analysis, unmeasured confounding could have affected some of the studies and therefore their pooled analysis, and finally publication bias, which may have provided only a partial view of the phenomenon studied, cannot be entirely ruled out.

A strength of our study is a clear distinction between disorder diagnosis and disorder symptoms in order to capture the relation between vitamin D status and the entire phenotypic continuum, thus avoiding the limitations of a purely binary classification (diagnosis yes/no). Additional strengths are the inclusion of mostly medium-high quality studies (as shown in Supplementary Tables S2 and S3), the identification of studies that established diagnoses according to the DSM or ICD and assessed symptoms with standardized tests, and the use of dose-response curve for studies investigating the relationship between vitamin D and ASD diagnosis.

## Conclusions

These findings confirm the beneficial association between higher maternal vitamin D levels and lower risk of ASD and ADHD in offspring and support the role of vitamin D in brain development and in neuroprotection.

## Implications for future clinical practice

Considering the properties of vitamin D and the emergence of many epidemiologic and experimental studies, it may be worthwhile to begin assessing vitamin D levels in early pregnancy and to consider appropriate supplementation, when needed to achieve adequate vitamin D levels. Such levels could favor neuronal proliferation, differentiation, and neuroprotection during the offspring’s brain development. This could represent a little protective component within the complex pathophysiology of ASD and ADHD, which involve numerous factors and mechanisms.

## Supplementary Information

Below is the link to the electronic supplementary material.

Supplementary Material 1 (DOCX 205 KB)

## Data Availability

The data that support the findings of this study is available from the corresponding author upon reasonable request.
