# Supplementary material for: The impact of maternal vitamin D levels during pregnancy and risk of autism spectrum disorder and attention deficit hyperactivity disorder diagnosis and symptoms in offspring: a systematic review and dose-response meta-analysis
Source: Eur Child Adolesc Psychiatry. 2026 Jun 3;35(7):2083–102. doi: 10.1007/s00787-026-03059-7 (PMC13427813; doi:10.1007/s00787-026-03059-7)
Supplement: Supplementary file 1 — Supplementary Material 1 (DOCX 205 KB) [file 787_2026_3059_MOESM1_ESM.docx]

**Supplementary Information**

**Supplementary Table S1.** Details of literature search on online databases with MeSH and non –MeSH terms

| **Database** | **Search string** | **Results (n)** |
| --- | --- | --- |
| PubMed | (((vitamin D[Title/Abstract]) OR (1 alpha, 25 dihydroxy 20 epi vitamin d3[MeSH Terms])) AND ((attention deficit disorders with hyperactivity[MeSH Terms]) OR (ADHD[Title/Abstract]) OR (autism spectrum disorder[MeSH Terms]) OR (autism spectrum disorders[Title/Abstract]) OR (child mental disorder[MeSH Terms]) OR (neurodevelopmental disorders[Title/Abstract]))) AND (child mental disorder[MeSH Terms]) | 418 |
| Web of Science | (TS=(vitamin d) OR TI=(vitamin d) OR AB=(vitamin d)) AND (TS=(attention deficit with hyperactivity) OR TI=(attention deficit with hyperactivity) OR AB=(attention deficit with hyperactivity) OR TS=(autism spectrum disorder) OR TI=(autism spectrum disorder) OR AB=(autism spectrum disorder) OR TS=(neurodevelopmental disorder) OR TI=(neurodevelopmental disorders) OR AB=(neurodevelopmental disorders)) | 699 |
| Embase | ('vitamin d':ti,ab OR 'vitamin d') AND ('adhd':ti,ab OR 'adhd' OR 'autism spectrum disorder':ti,ab OR 'autism spectrum disorder' OR 'neurodevelopmental disorders':ti,ab OR 'neurodevelopmental disorders') | 667 |
| Cochrane | (vitamin d):ti,ab,kw AND (("ADHD"):ti,ab,kw OR ("autism spectrum disorder"):ti,ab,kw OR ("neurodevelopmental disorders"):ti,ab,kw) | 99 |

**Supplementary Table S2.** Newcastle - Ottawa quality assessment scale for included studies with **cohort and case-cohort design** (A), with details of score assignment with number of stars in parenthesis (B)

| **(A) Newcastle - Ottawa quality assessment scale for cohort and case-cohort studies** | | | | | | | | | | |
| --- | --- | --- | --- | --- | --- | --- | --- | --- | --- | --- |
| **Selection**  1) Representativeness of the exposed cohort   1. truly representative of the average **developmental age population** (<18) in the community* 2. somewhat representative of the average **developmental age population** (<18) in the community 3. selected group of users e.g. nurses, volunteers 4. no description of the derivation of the cohort   2) Selection of the non-exposed cohort   1. drawn from the **same** community as the exposed cohort* 2. drawn from a different source 3. no description of the derivation of the non-exposed cohort   3) Ascertainment of exposure   1. secure record (vitamin D levels on maternal serum/neonatal cord or blood spot)***** 2. structured interview***** 3. written self-report 4. no description   4) Demonstration that outcome of interest was not present at start of study   1. yes* 2. no | | | | | **Comparability**  1) Comparability of cohorts on the basis of the design or analysis   1. study controls for **child’s age and sex at assessment and for maternal age*** 2. study controls for **seasonality of collection or maternal socioeconomic status/education level*** | | | | | |
|  |  |  |  |  | **Outcome**  1) Assessment of outcome   1. independent blind assessment/**diagnosis and evaluation or symptoms obtained through standardized questionnaires*** 2. record linkage*? 3. self-report 4. no description   2) Was follow-up long enough for outcomes to occur   1. yes (**5 years** follow up period has considered adequate)***** 2. no   3) Adequacy of follow up of cohorts   1. complete follow up accounted for **100% of subjects*** 2. subjects lost to follow up unlikely to introduce bias – **lost at follow-up ≤20%*** 3. follow up rate **<80%** 4. no statement | | | | | |
| **(B) Details of score assignment** | | | | | | | | | | |
| **Reference** | ***Selection*** | | | | ***Comparability*** | | ***Outcome*** | | | ***Total*** |
|  | *S-1* | *S-2* | *S-3* | *S-4* | *C-1* | *C-2* | *O-1* | *O-2* | *O-3* | ***Score*** |
| Horsdal 2025 | a (1) | a (1) | a (1) | a (1) | a (1) | b (1) | a (1) | a (1) | b (1) | 9 |
| Lee 2019 | a (1) | a (1) | a (1) | a (1) | a (0) | b (1) | a (1) | b (0) | a (1) | 7 |
| Lòpez-Vicente 2019 | a (1) | a (1) | a (1) | a (1) | a (0) | b (1) | a (1) | a (1) | c (0) | 7 |
| Madley-Dowd 2022 | a (1) | a (1) | a (1) | a (1) | a (0) | b (1) | a (1) | a (1) | c (0) | 7 |
| van Roij 2025 | a (1) | a (1) | a (1) | a (1) | a (0) | b (0) | a (1) | a (1) | c (0) | 6 |
| Morales 2015 | a (1) | a (1) | a (1) | a (1) | a (0) | b (1) | a (1) | b 0) | c (0) | 6 |
| Daraki 2017 | a (1) | a (1) | a (1) | a (1) | a (0) | b (1) | a (1) | b (0) | b (1) | 7 |
| Thinggaard 2024 | a (1) | a (1) | a (1) | a (1) | a (1) | b (1) | a (1) | a (1) | b (1) | 9 |

**Supplementary Table S3.** Newcastle - Ottawa quality assessment scale for included studies with **case-control design** (A), with details of score assignment with number of stars in parenthesis (B)

| **(A) Newcastle - Ottawa quality assessment scale for case-control studies** | | | | | | | | | | |
| --- | --- | --- | --- | --- | --- | --- | --- | --- | --- | --- |
| **Selection**  1) Is the case definition adequate?   1. yes, registry-based or with independent validation* 2. yes but based on self-reports 3. no sufficient details or no description   2) Representativeness of the cases   1. consecutive or obviously representative series of cases* 2. potential for selection biases or not stated   3) Selection of Controls   1. community controls* 2. hospital controls 3. no description   4) Definition of Controls   1. no history of disease (endpoint)* 2. no description of source | | | | | **Comparability**  1) Comparability of cases and controls on the basis of the design or analysis   - 1. study controls for **child’s age and sex at assessment and for maternal age***   2. study controls for **seasonality of collection or maternal socioeconomic status/education level*** | | | | | |
|  |  |  |  |  | **Exposure**  1) Ascertainment of exposure   1. secure record (**vitamin D levels on maternal serum/neonatal cord or blood spot**)* 2. structured interview where blind to case/control status* 3. interview not blinded to case/control status 4. written self-report or medical record only 5. no description   2) Same method of ascertainment for cases and controls   1. yes* 2. no   3) Non-Response rate   1. same rate for both groups* 2. non respondents described 3. rate different and no designation | | | | | |
| **(B) Details of score assignment** | | | | | | | | | | |
| **Reference** | ***Selection*** | | | | ***Comparability*** | | ***Exposure*** | | | ***Total*** |
|  | *S-1* | *S-2* | *S-3* | *S-4* | *C-1* | *C-2* | *E-1* | *E-2* | *E-3* | ***Score*** |
| Chen 2016 | a (1) | a (1) | a (1) | a (1) | a (1) | b (0) | a (1) | a (1) | c (0) | 7 |
| Egorova 2020 | a (1) | a (1) | a (1) | a (1) | a (0) | b (0) | a (1) | a (1) | c (0) | 6 |
| Schmidt 2019 | a (1) | a (1) | a (1) | a (1) | a (0) | b (1) | a (1) | a (1) | a (1) | 8 |
| Sourander 2021 | a (1) | a (1) | a (1) | a (1) | a (0) | b (1) | a (1) | a (1) | a (1) | 8 |
| Windham 2020 | a (1) | a (1) | a (1) | a (1) | a (1) | b (1) | a (1) | a (1) | a (1) | 9 |
| Gustafsson 2015 | a (1) | a (1) | a (1) | a (1) | a (0) | b (1) | a (1) | a (1) | a (1) | 8 |
| Sucksdorff 2021 | a (1) | a (1) | a (1) | a (1) | a (0) | b (1) | a (1) | a (1) | a (1) | 8 |

**Supplementary Fig. 1** Funnel plots of the studies related to the association between maternal vitamin D levels and offspring ASD diagnosis. SE: Standard Error, RR: Risk Ratio, CI: Confidence Interval


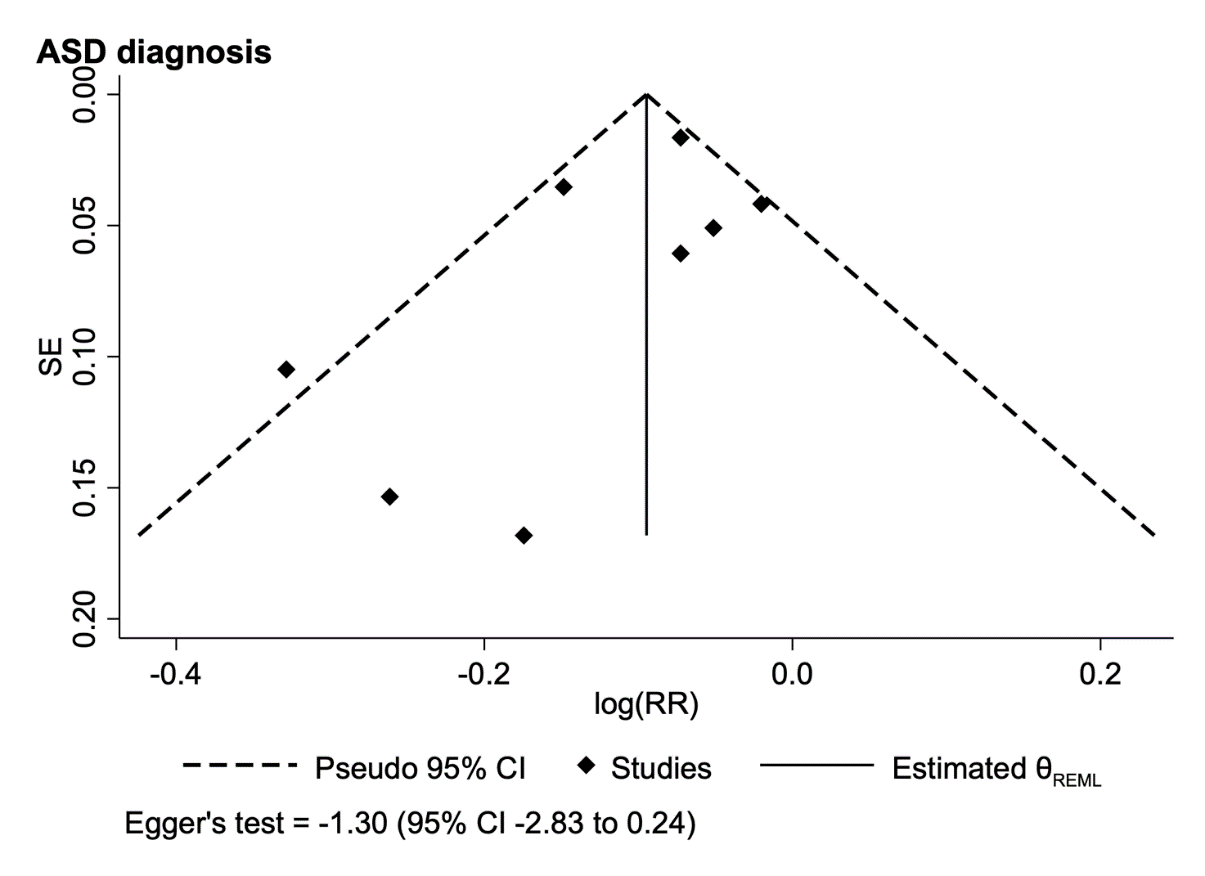


**Supplementary Fig. 2** Funnel plots of the studies related to the association between maternal vitamin D levels and offspring ASD diagnosis with adjusted estimate. SE: Standard Error, RR: Risk Ratio, CI: Confidence Interval


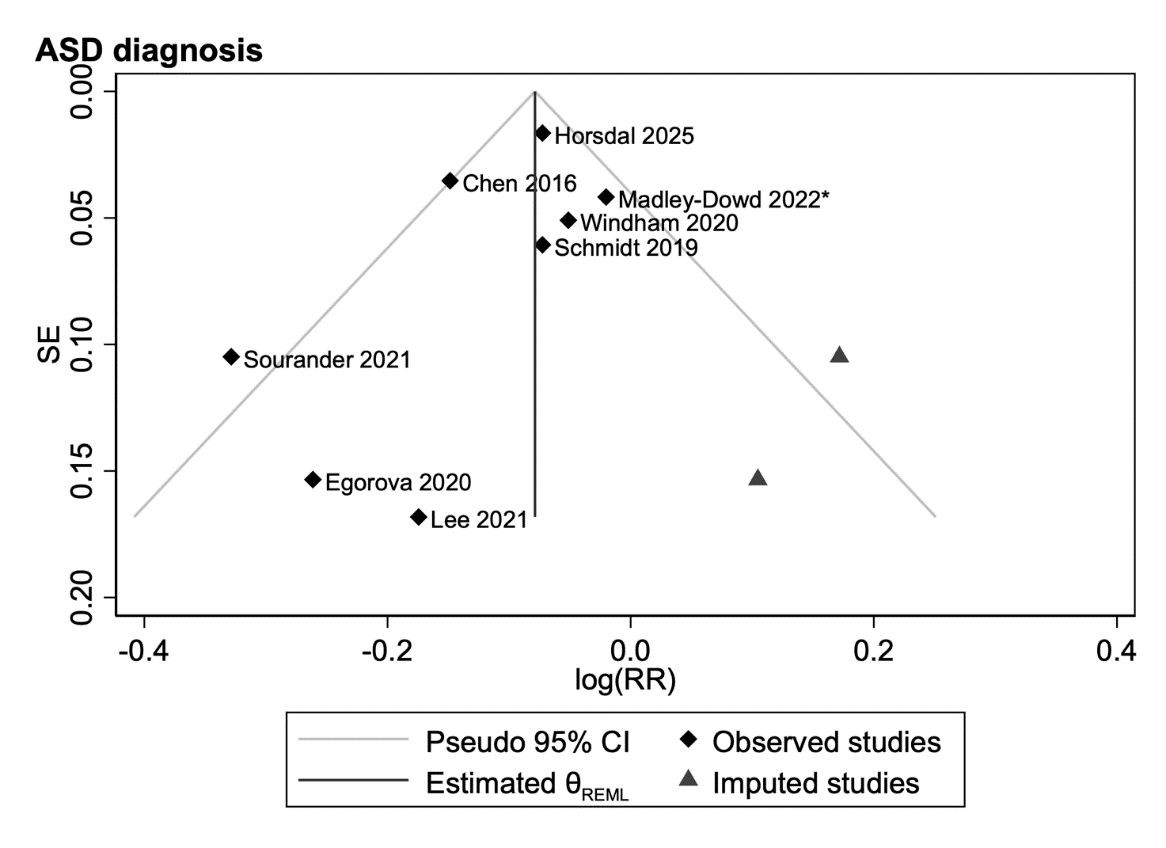


**Supplementary Fig. 3** Dose-response meta-analysis according to increasing maternal vitamin D exposure concentrations and risk of offspring ASD diagnosis. Spline curve (solid black line) with 95% confidence limits (grey area) and specific dose-response relationship of each study (solid grey lines). RR: Risk Ratio

**
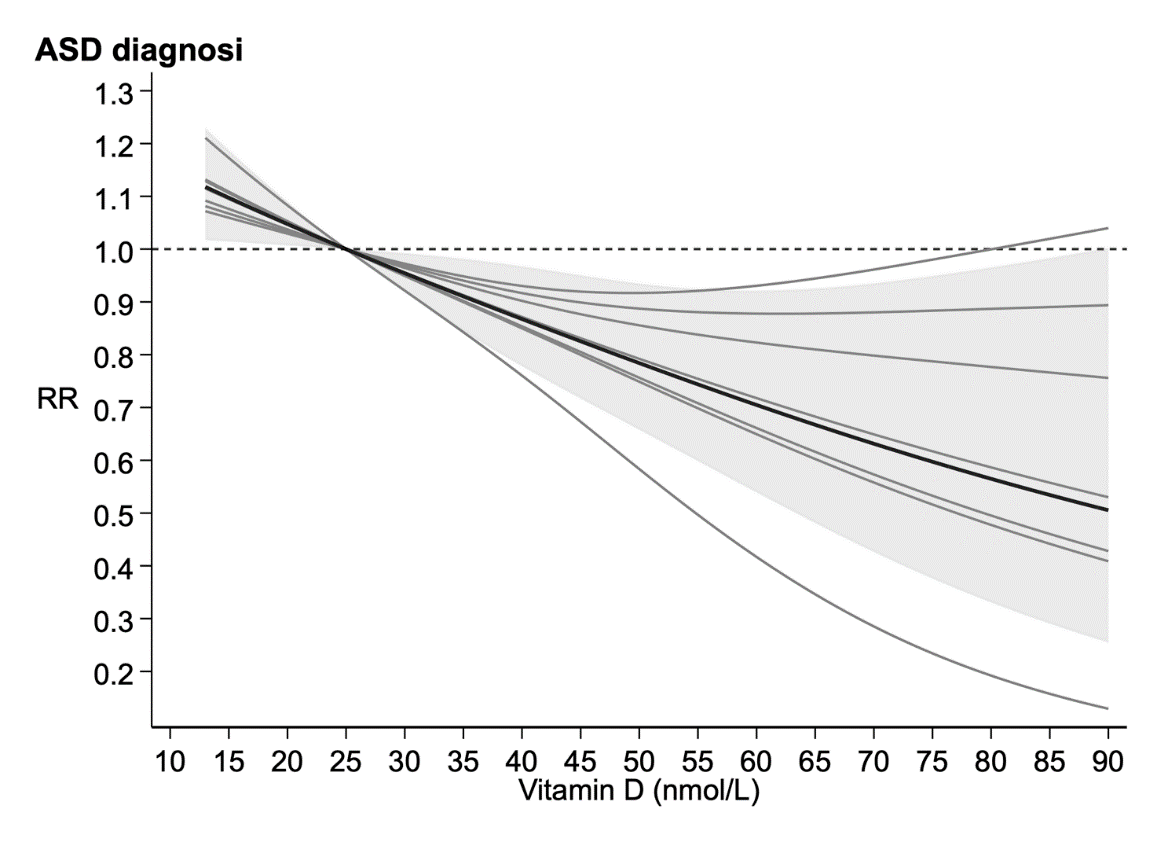
**
